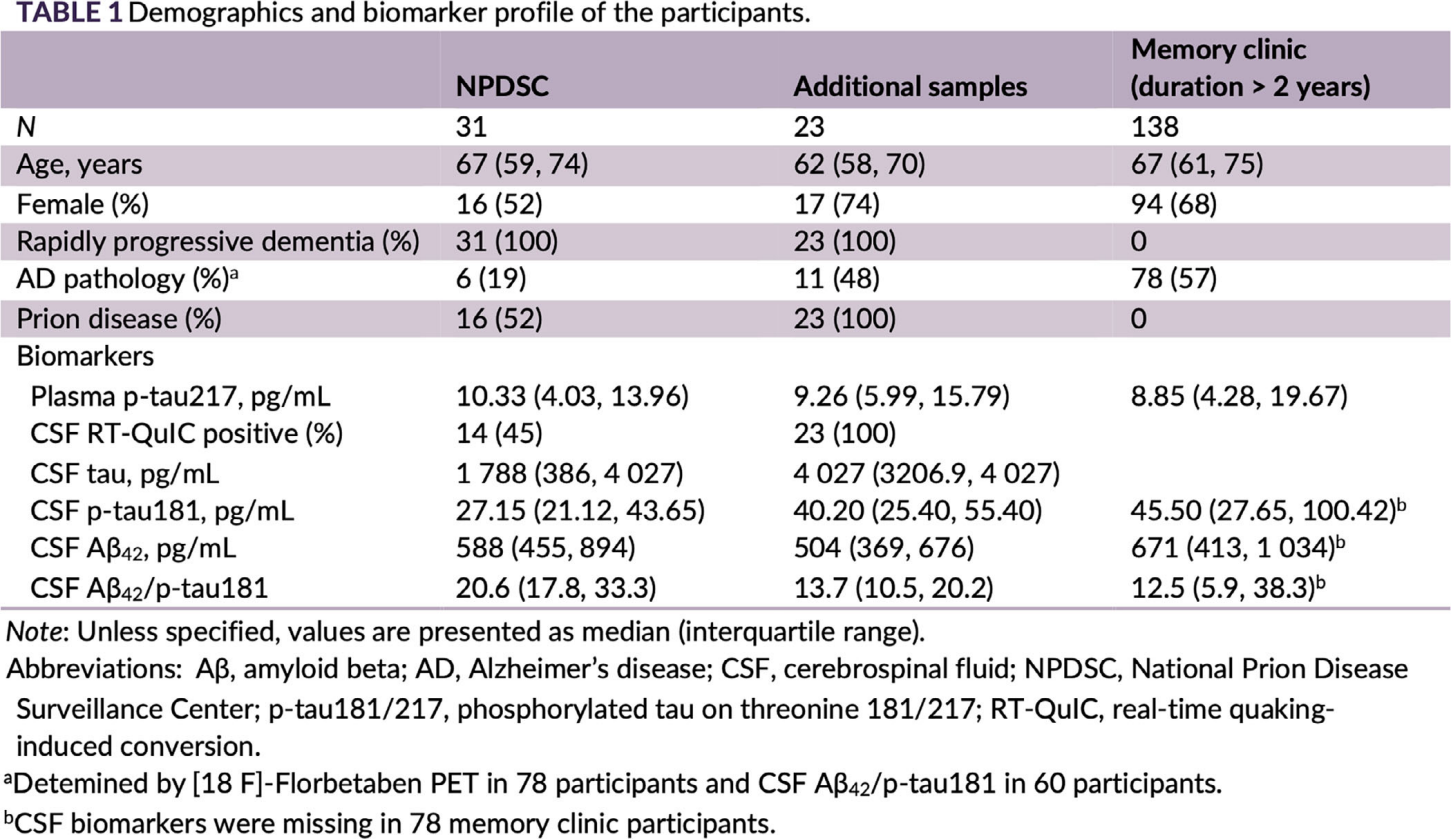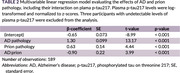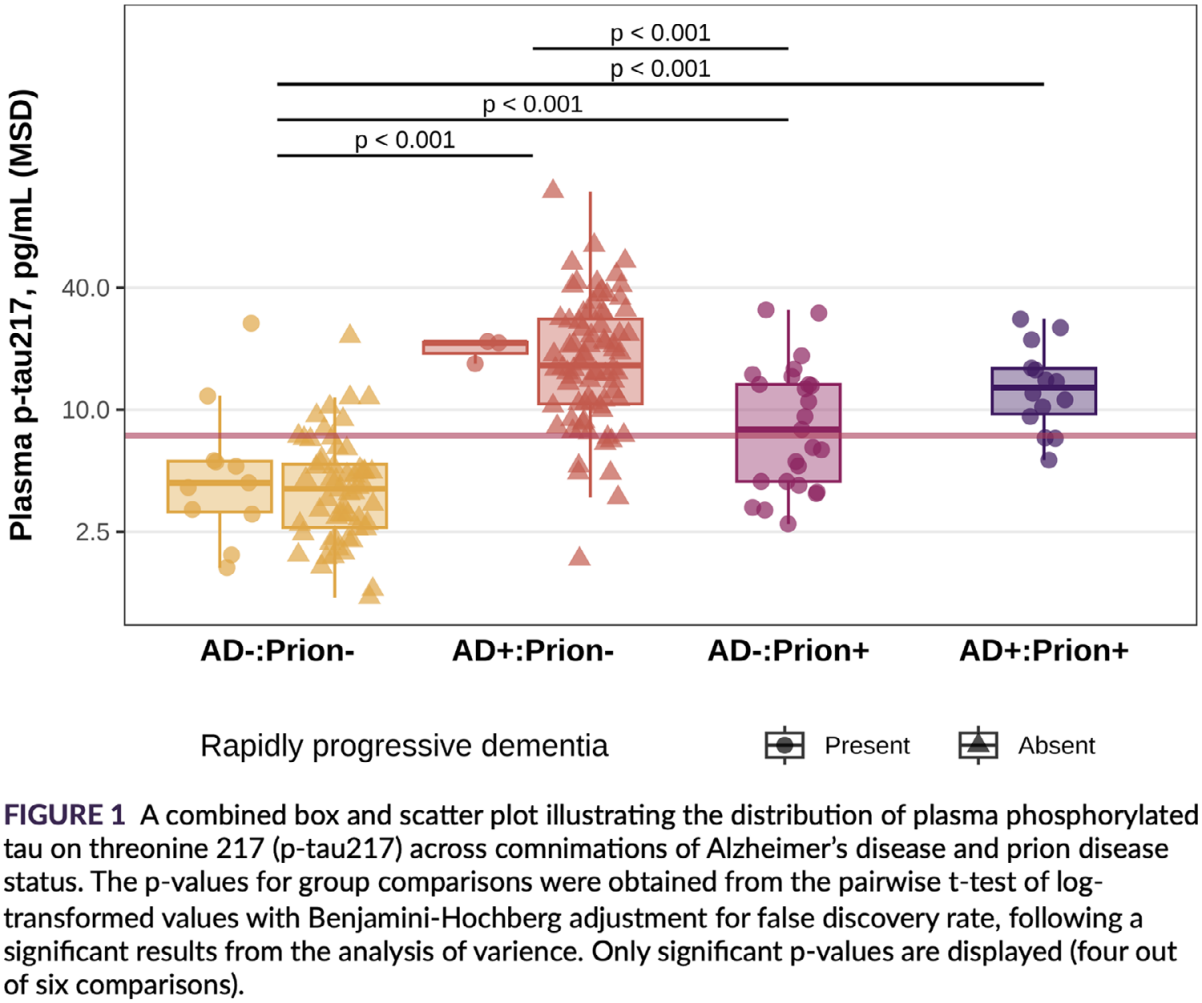# Plasma *p*‐tau217 in individuals with prion and Alzheimer's pathology: diagnostic caveats in rapidly progressive dementias

**DOI:** 10.1002/alz70856_106353

**Published:** 2026-01-08

**Authors:** Poosanu Thanapornsangsuth, Adipa Chongsuksantikul, Watayuth Luechaipanit, Thanaporn Haethaisong, Prawit Oangkhana, Kittithatch Booncharoen, Jedsada Khieukhajee, Yuttachai Likitjaroen, Chayanis Yolsiriwat, Abhinbhen Wasontiwong Saraya

**Affiliations:** ^1^ Memory Clinic, King Chulalongkorn Memorial Hospital, The Thai Red Cross Society, Bangkok, Thailand; ^2^ Thai Red Cross Emerging Infectious Diseases Health Science Centre, King Chulalongkorn Memorial Hospital, Bangkok, Thailand; ^3^ Division of Neurology, Department of Medicine, Faculty of Medicine, Chulalongkorn University, Bangkok, Thailand; ^4^ Thai Red Cross Emerging Infectious Diseases Health Science Centre, King Chulalongkorn Memorial Hospital, The Thai Red Cross Society, Bangkok, Thailand; ^5^ Neurology Center, Phyathai 1 Hospital, Bangkok, Rachathewi, Thailand; ^6^ Neurocognitive Unit, Division of Neurology, Department of Medicine, Faculty of Medicine, Chulalongkorn University, Bangkok, Thailand; ^7^ Neurological Institute of Thailand, Ratchathewi, Bangkok, Thailand

## Abstract

**Background:**

Plasma *p*‐tau217 is widely regarded as a specific biomarker for Alzheimer's disease (AD). However, recent studies have reported substantial elevations in prion disease, suggesting prion‐induced *p*‐tau release. This study investigates the relationship between plasma *p*‐tau217 and AD/prion pathology and examines its diagnostic implications in rapidly progressive dementia (RPD), where prion disease is predominant but rapidly progressive AD is also significant.

**Method:**

Participants were from three sources: (1) the Thai National Prion Disease Surveillance Center (NPDSC), a prospective registry of suspected prion disease cases (March 2023–December 2024); (2) additional CSF from RT‐QuIC–positive patients across Thailand (March 2022–June 2024); and (3) memory clinic patients with disease duration >2 years from King Chulalongkorn Memorial Hospital and the Neurological Institute of Thailand (2022–October 2024). AD pathology was confirmed by CSF amyloid‐β42/p‐tau or florbetaben PET. Prion disease was defined by positive CSF RT‐QuIC or MRI criteria for “Definitely CJD”, absence of an alternative diagnosis, and death within six months. Memory clinic patients were presumed prion‐negative based on prolonged disease duration (>2 years) and negative MRI. A multivariable linear regression model assessed the effects of AD and prion pathology, including their interaction. Pairwise comparisons were adjusted using Benjamini‐Hochberg correction. The diagnostic accuracy of plasma *p*‐tau217 for AD, prion disease, or both was evaluated using ROC analysis in the prospectively‐enrolled NPDSC participants.

**Result:**

A total of 192 participants were included (31 NPDSC, 23 RT‐QuIC–positive, 138 memory clinic, Table 1). Both AD and prion pathology were individually associated with increased plasma *p*‐tau217. Interestingly, an interaction effect was observed, with lower plasma *p*‐tau217 levels in individuals with both AD and prion disease compared to AD alone, demonstrating non‐linearity or even decreasing effects on plasma *p*‐tau217 (Table 2 and Figure 1). Plasma *p*‐tau217 showed poor‐to‐moderate accuracy for AD (AUC 0.76, 95% CI 0.58–0.94), prion disease (AUC 0.63, 95% CI 0.42–0.85), and for detecting any pathology (AUC 0.79, 95% CI 0.62–0.97).

**Conclusion:**

Plasma *p*‐tau217 is not specific for AD, as it is elevated in prion disease irrespective of AD pathology. These findings highlight its limited diagnostic utility in RPD. The interaction between pathologies in modulating plasma *p*‐tau217 warrants further investigation.